# Exploring the association between patient‐drawn pain diagrams and psychological and physical health variables: A large‐scale study of patients with low back pain

**DOI:** 10.1002/ejp.4711

**Published:** 2024-08-07

**Authors:** Steen Harsted, Natalie H. S. Chang, Casper Nim, James J. Young, David T. McNaughton, Søren O'Neill

**Affiliations:** ^1^ Department of Sports Science and Clinical Biomechanics, Center for Muscle and Joint Health University of Southern Denmark Odense M Denmark; ^2^ Medical Spinal Research Unit, Spine Centre of Southern Denmark University Hospital of Southern Denmark Middelfart Denmark; ^3^ Department of Regional Health Research University of Southern Denmark Odense M Denmark; ^4^ Schroeder Arthritis Institute, Krembil Research Institute University Health Network Toronto Canada; ^5^ School of Psychological Sciences Macquarie University Sydney New South Wales Australia

## Abstract

**Background:**

Despite the use of Patient‐Drawn Pain Drawings (PDPDs) in clinical settings, their validity as indicators of psychological distress remains debated. We aimed to assess the association between PDPD areas and physical health and psychological variables.

**Methods:**

This study analysed digitally‐drawn PDPDs from 15,345 chronic low back pain (LBP) patients at a Danish outpatient hospital unit. We employed a novel quantitative approach to calculate four log‐transformed geometric pain areas for each PDPD. We assessed six psychological constructs and seven physical health variables. Associations were modelled using multivariable linear regression.

**Results:**

Increasing leg pain intensity (estimates from 0.12 to 0.25), disability (estimates from 0.3 to 0.14), and pain duration (estimates from 0.10 to 0.33) had the strongest associations with increasing pain areas. Conversely, increasing fear of movement (estimates from −0.02 to −0.05) and catastrophizing (estimates from −0.02 to −0.03) were associated with slight reductions in pain areas. Anxiety and depression had the weakest and most uncertain relationships to pain area size.

**Conclusions:**

Increasing levels of leg pain intensity, pain duration, and pain‐related disability were consistently associated with larger geometric pain areas in PDPDs. Conversely, the associations between the psychological constructs and the geometric pain areas exhibited varying directions and were notably weaker. Clinicians are encouraged to focus on the association of PDPDs with physical symptoms rather than psychological conditions during clinical assessments.

**Significance Statement:**

This large‐scale study demonstrates that extensive pain areas in pain drawings drawn by LBP patients do not signify psychological distress. Our findings reveal that these pain representations are more closely linked to increased pain intensity, pain duration, and disability rather than being independently associated with psychological factors. Clinicians are encouraged to focus on the association of extensive pain areas with physical symptoms rather than psychological distress during clinical assessments.

## 1 INTRODUCTION

Low back pain (LBP) is a leading cause of global disability, with a significant proportion of individuals experiencing persistent and disabling symptoms (Ferreira et al., 2023). LBP's biopsychosocial framework highlights a complex interplay in symptom variability (Fillingim, 2017; Hassan et al., 2023). Importantly, non‐favourable phenotypes, which may include high psychological distress and high pain‐related disability, have been associated with a worse prognosis and higher healthcare utilization costs (Mutubuki et al., 2020). Identifying valid, brief, and practical assessment methods for non‐favourable LBP phenotypes is essential across healthcare levels.

Patient‐Drawn Pain Drawings (PDPDs) are an easily implementable, valid, and reliable assessment technique widely used in clinical and research settings to depict the subjective experience of somatic pain, including its location and distribution (Schott, 2010; Shaballout, Aloumar, et al., 2019; Southerst et al., 2013). PDPDs may aid in classifying non‐favourable LBP phenotypes, potentially identifying patients with worse psychological distress and a poor prognosis (Mirkhil & Kent, 2009; Shaballout et al., 2019b). Historically, since the 1970s, larger areas in PDPDs have been interpreted as indicative of increased psychological distress or complex psychological presentations. This concept, described as ‘magnification’ or ‘expansion’ by Ransford et al. (1976), was thought to be particularly evident in drawings that extend beyond the defined body outline (Ransford et al., 1976). Udén et al. (1988) further distinguished between organic and non‐organic drawings, characterizing non‐organic patterns as poorly defined or bizarrely expanded, often non‐anatomical in appearance (Udén et al., 1988). These historical perspectives on PDPDs were echoed in Gordon Waddell's influential book ‘The Back Pain Revolution,’ which also discussed psychological inferences from pain drawings. The book highlighted cases of disc prolapse patients where extensive pain areas in drawings were interpreted as manifestations of psychological distress, further popularizing the concept of ‘magnification’ in clinical assessment (Waddell, 1999). Previous research has established a consistent relationship between increasing somatic pain sites, identified from PDPDs, with greater anxiety, depression, catastrophizing, pain‐related disability, and fear of movement (Alter et al., 2021; Nordstoga et al., 2017). However, the interpretation of pain drawing patterns as indicators of psychological distress remains controversial. Two systematic reviews have concluded that the available evidence does not support the use of pain drawings as a reliable tool for inferring psychological distress (Bertozzi et al., 2015; Carnes et al., 2006). This conclusion may be premature, considering that the reviews are based on studies with relatively small sample sizes—the largest study included only 651 patients—and varied methodologies for quantifying pain drawings. Furthermore, despite these systematic reviews, primary and secondary care clinicians continue to perceive a link between pain drawings and psychological factors. This divergence between clinical practice and research findings underscores the need for further empirical investigation with results presented in a format both informative and easily interpretable by clinicians.

Our study aims to address past research limitations by employing a novel quantitative method in a large LBP patient sample to analyse geometric pain areas in PDPDs, offering new insights into LBP symptom assessment (O'Neill et al., 2019). This method obtains reliable and valid metrics of the total area of pain drawn, the area drawn inside the pain drawing silhouette, the area drawn outside the pain drawing silhouette, and the number of pain regions indicated, and offers a novel perspective on the assessment of pain areas.

Further, given the contested utility of PDPDs as an indicator of high psychological distress and/or pain‐related disability, our primary objective is to determine the association between PDPD areas and physical health and psychological variables in a large sample of patients with persistent LBP. In doing so, we will shed light on the potential utility of PDPDs as a proxy for psychological and physical health in patients with persistent LBP.

## 2 METHODS

### 2.1 Study design

We conducted a cross‐sectional analysis with data collected from the Spine Centre of Southern Denmark, where all patients completed the SpineData registry before their initial consultation (Kent et al., 2015). SpineData is regulated as information from electronic patient records. However, as the patients are not currently treated and we can obtain the data anonymously, there is no need to obtain ethical approval for this study according to Danish law. The Danish Act on Research Ethics Review of Health Research Projects regulates data processing in section 14, subsection 2.

Our findings are reported according to Strengthening the Reporting of Observational Studies in Epidemiology (STROBE) (von Elm et al., 2008).

### 2.2 Participants

Participants were included from the Spine Centre of Southern Denmark, an outpatient public‐funded hospital unit specializing in adult spine pain syndromes. General practitioners, chiropractors, and other hospital departments can refer patients to the centre after unsuccessful treatment in primary care (e.g. physiotherapy or chiropractic practice). The centre consists of both a medical and surgical unit and sees a diverse patient population ranging from acute disc herniations with radiculopathy to complex chronic pain syndromes. From February 2019 to April 2021, 16,239 patients with LBP were issued the questionnaire. All patients aged 18 and above who completed the PDPDs were included in our study.

### 2.3 Baseline characteristics

#### 2.3.1 Age, sex and body mass index

We calculated age and sex based on the patient's social security number. Sex was scored as binary (male/female). Body mass index (BMI) was calculated based on self‐reported height and weight (numeric values).

#### 2.3.2 Pain‐related disability

The patients filled out the Danish version of the Oswestry disability index (Lauridsen et al., 2006) by scoring 10 items on a five‐point Likert scale; the scoring goes from 0% (no disability) to 100% (completely disabled). We scaled this to a 0–10 score (numeric value) for ease of comparison.

#### 2.3.3 LBP intensity

LBP intensity was scored by the patients using a numerical rating scale for current pain intensity on a 0 (no pain) to 10 (worst imaginable pain) score (numeric value) (Manniche et al., 1994).

#### 2.3.4 Leg pain intensity

The patients scored their leg pain intensity using a numerical rating scale for current pain intensity on a 0 (no pain) to 10 (worst imaginable pain) score (numeric value) (Manniche et al., 1994).

#### 2.3.5 Pain duration

The patients were asked to specify the onset date of their pain. The duration of pain was calculated as the time interval between the onset and data collection dates.

#### 2.3.6 Psychological measures

Psychological measures were measured using questions from the Brief Screening Questions questionnaire and the short form of the Örebro Musculoskeletal Pain Screening Questionnaire (Kent et al., 2014; Linton et al., 2011). These questionnaires have demonstrated robust concurrent validity with full‐length questionnaires across various psychosocial constructs, including anxiety, depression, social isolation, catastrophization, and fear of movement. Specifically, they have shown correlations ranging from 0.62 to 0.95 with validated full‐length questionnaires, high overall accuracy (78%–93%), and stable results across different demographics and pain characteristics (Kent et al., 2014; Linton et al., 2011). Six constructs were scored by the patients using a scale going from 0 (no distress) to 10 (high distress) (numeric value). The six constructs were: Perceived risk of persistent pain (single item), Fear of movement (mean of two items), Anxiety (single item), Catastrophizing (mean of two items), Depression (mean of two items), and Loneliness (single item). The questions are available in the supplementary material.

#### 2.3.7 Pain drawing data

When completing the SpineData questionnaire, patients are presented with a digital pain drawing onto which they can draw on both touch screens and regular computer screens using a computer mouse. The interface is accompanied by the instruction in Danish: ‘Brug din finger eller mus til at tegne, hvor dine smerter er,’ which translates to ‘Use your finger or mouse to draw where your pain is.’ The pain drawing interface was programmed with a fixed stroke width of 5 pixels. Some patients completed the questionnaire on a digital tablet in the waiting room, but the majority did so online in a 48‐hour window prior to the first consultation.

The pain drawing interface stores data as scalable vector graphics (SVG). Data consists of coordinate points in a Cartesian coordinate system (450 × 500 pixels) for each ‘stroke’ the patient draws. A stroke thus defines a continuous path from a start point (‘pen‐down) to a finish point (‘pen‐up). In this manner, each pain drawing consists of a variable number of strokes, and each stroke consists of a variable number of points in the coordinate system.

Geometric quantification of the drawn areas was done by computer algorithms programmed in the statistical computing language R. The algorithms are briefly described here; further details are available elsewhere (O'Neill et al., 2019).

#### 2.3.8 Total area

Each individual stroke was converted into a polygon by linking the first and last coordinate points. Overlapping polygons were then merged to avoid double‐counting any areas. The total geometric area of each individual's pain drawing was subsequently calculated by summing all the areas of these polygons.

#### 2.3.9 Area inside and area outside the pain drawing Silhouette

In order to measure the area of pain indicated inside or outside the body silhouette, a polygon was constructed to match the silhouette on the pain drawing background. The Area Inside was subsequently determined by calculating the intersection between this polygon and the Total Area polygon. Conversely, the Area Outside was measured by calculating the remaining area that fell outside of this intersection.

#### 2.3.10 Number of regions with pain

The body silhouette was divided into 46 anatomical regions during data processing. For each region, a binary variable was assigned, indicating whether the patients' drawing involved that specific area. The “Number of Regions with Pain” was subsequently computed as the non‐weighted count of involved regions.

### 2.4 Statistical analysis

#### 2.4.1 Data transformation

We examined the distribution of all variables graphically using density plots and histograms. Non‐normally distributed variables were considered for log transformation or reclassification as categorical variables. Consequently, the four area measures underwent log transformation for analysis (see Data S1). When the ‘Area Outside’ measure contained zero values, these were recoded to 0.1 to prevent undefined values during the log transformation. Due to small variances in the loneliness score, as most participants (58%) scored 0, we dichotomized this into ‘no loneliness’ (score of 0) or ‘experienced loneliness’ (score equal to or larger than 1) (see Data S1). Pain duration was dichotomized into two categories: ‘1 year or less’ and ‘more than 1 year’. This dichotomization was guided by the observed distribution of pain duration values within the patient cohort (see Data S1).

#### 2.4.2 Descriptive statistics and plots

Descriptive statistics were used to summarize all variables: counts and percentages were used for categorical variables, while continuous variables were described using means and standard deviations if normally distributed or medians and interquartile ranges if not.

To visualize the distribution of the raw pixel coordinates from the pain drawings across distinct categories of the independent variables, 1000 randomly selected PDPDs for each category were superimposed using a high transparency setting. This allowed for a feasible and easy‐to‐read visual comparison of pain locations and distributions within and across groups. Right‐sided coordinates from unilateral pain distributions were flipped to the left side to facilitate comparisons, as this approach enabled us to visualize and differentiate between uni‐ and bilateral pain distributions more effectively.

#### 2.4.3 Regression analysis

The relationships between the independent variables and the four area measures were examined first using univariable linear regression models and then repeated using four full multivariable linear regression models. Tests of model assumptions were based on the normality of residuals inspected via density plots and quantile‐quantile plots and homoscedasticity via residual‐versus‐fitted values plots. In all multivariable models, only variables with a variance inflation factor of less than 5 were included to avoid multicollinearity. Associations were reported as beta estimates with 95% confidence intervals (CI). Cases with missing values were omitted from the analysis using listwise deletion, consistent with the default behaviour for linear regression modelling.

#### 2.4.4 Sensitivity analysis

We examined the relationship between the ‘Area outside’ measure and the independent variables using a range of regression models other than multivariable linear regression to manage zero‐inflation and overdispersion in the data. These models included zero‐inflated negative binomial regression, Poisson regression, and ordinal logistic regression.

All statistical analyses were performed using R (vers.4.3) (R Core Team, 2023), RStudio (Build 446), and the R packages tidyverse (Wickham et al., 2019), gtsummary (Sjoberg et al., 2020), Ggally (Schloerke et al., 2020), performance (Lüdecke et al., 2021), and magick (Ooms, 2023).

## 3 RESULTS

Of the 16,239 LBP patients who completed SpineData during the study period, 15,345 (94.5%) provided pain drawings and were aged 18 or older, and thus constituted our study sample. The participants had a mean age of 55.2 years (SD 16.4), a mean BMI of 27.8 (SD 5.4), and 55% were female. Missing values accounted for less than 4% of the total dataset for any given variable. Further descriptive statistics for this population are presented in Table 1.

**TABLE 1:** Descriptive statistics.

	*N*	*N* = 15,345^a^
Age (Years)	15,345	55.2 (16.4)
Sex	15,345	
Male		6880 (45%)
Female		8465 (55%)
Body mass index	15,101	27.8 (5.4)
Pain related disability	14,780	3.6 (1.7)
Pain intensity back	15,143	6.0 (4.0, 7.0)
Pain intensity leg	15,076	5.0 (2.0, 7.0)
Pain duration	14,760	
1 year or less		7822 (53%)
More than 1 year		6938 (42%)
Perceived risk of chronicity	14,758	8.0 (5.0, 9.0)
Loneliness	14,921	
No (Social score = 0)		8690 (58%)
Yes (Social score >0)		6231 (42%)
Fear of movement	14,713	5.0 (2.0, 7.0)
Catastrophizing	14,921	5.0 (2.5, 7.5)
Anxiety	14,915	4.0 (1.0, 7.0)
Depression	14,902	4.5 (1.5, 7.0)
Area total (log changed)	15,345	6.8 (1.8)
Area inside (log changed)	15,345	6.7 (1.7)
Area outside (log changed)	15,345	2.2 (−2.3, 5.2)
Area as nr. of regions with pain (log changed)	15,345	1.9 (0.7)

^a^ Mean (SD); *n* (%); Median (IQR).

**FIGURE 1:**
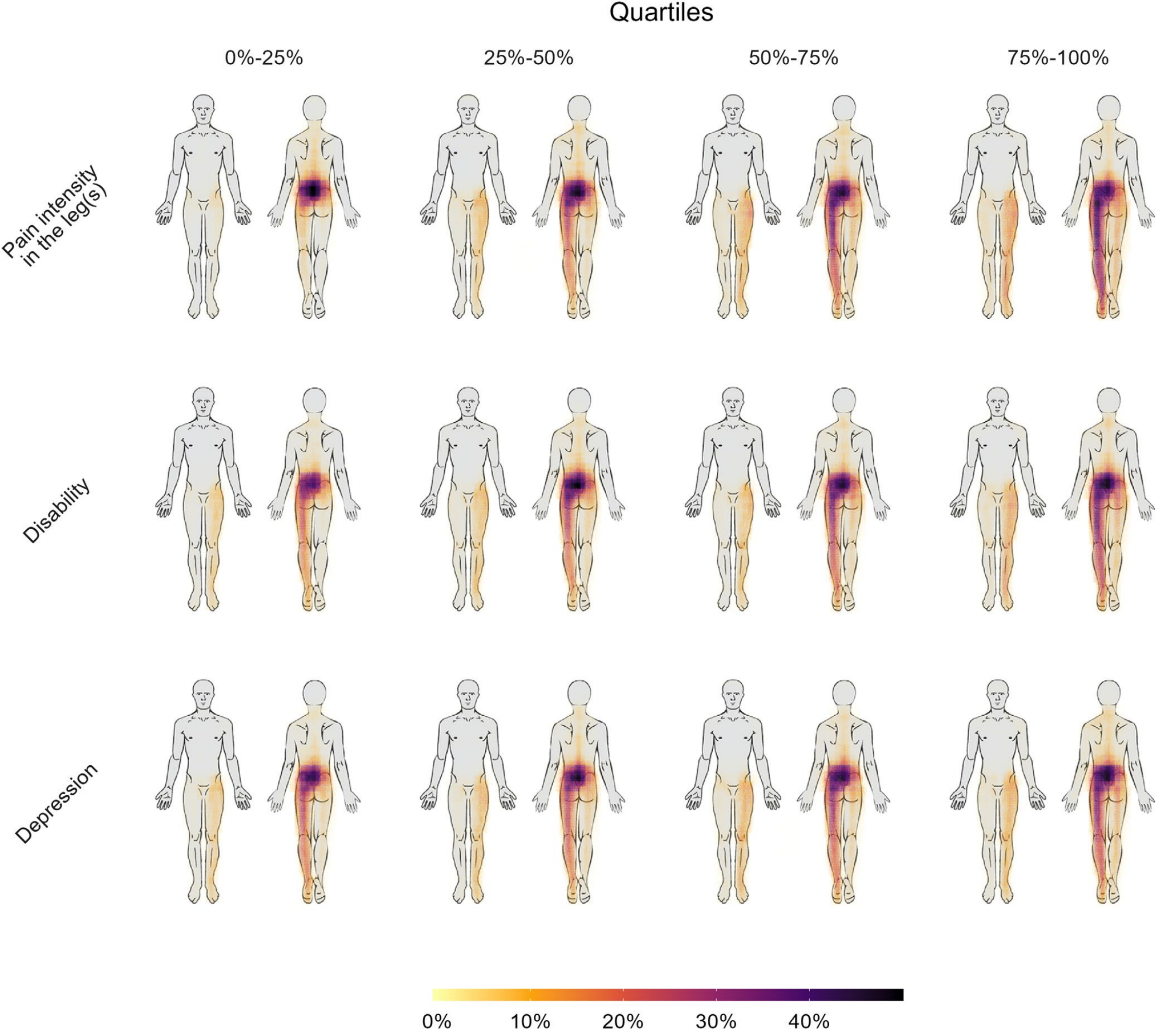
Aggregate pain drawings. Each cell in the 3 × 4 grid represents a high transparency overlay of 1000 pain drawings. Unilateral pain is consistently depicted on the left side of the body. Each row categorizes 4000 patients into four quartiles based on their respective scores in pain intensity in the legs, disability, and depression. The quartiles range from the lowest (leftmost column) to the highest (rightmost column) scores. The colour gradient represents the percentage of patients within each cell who drew their pain in specific body areas. Higher scores are associated with larger pain areas across all three variables, but are most notable for pain intensity in the legs.

**FIGURE 2:**
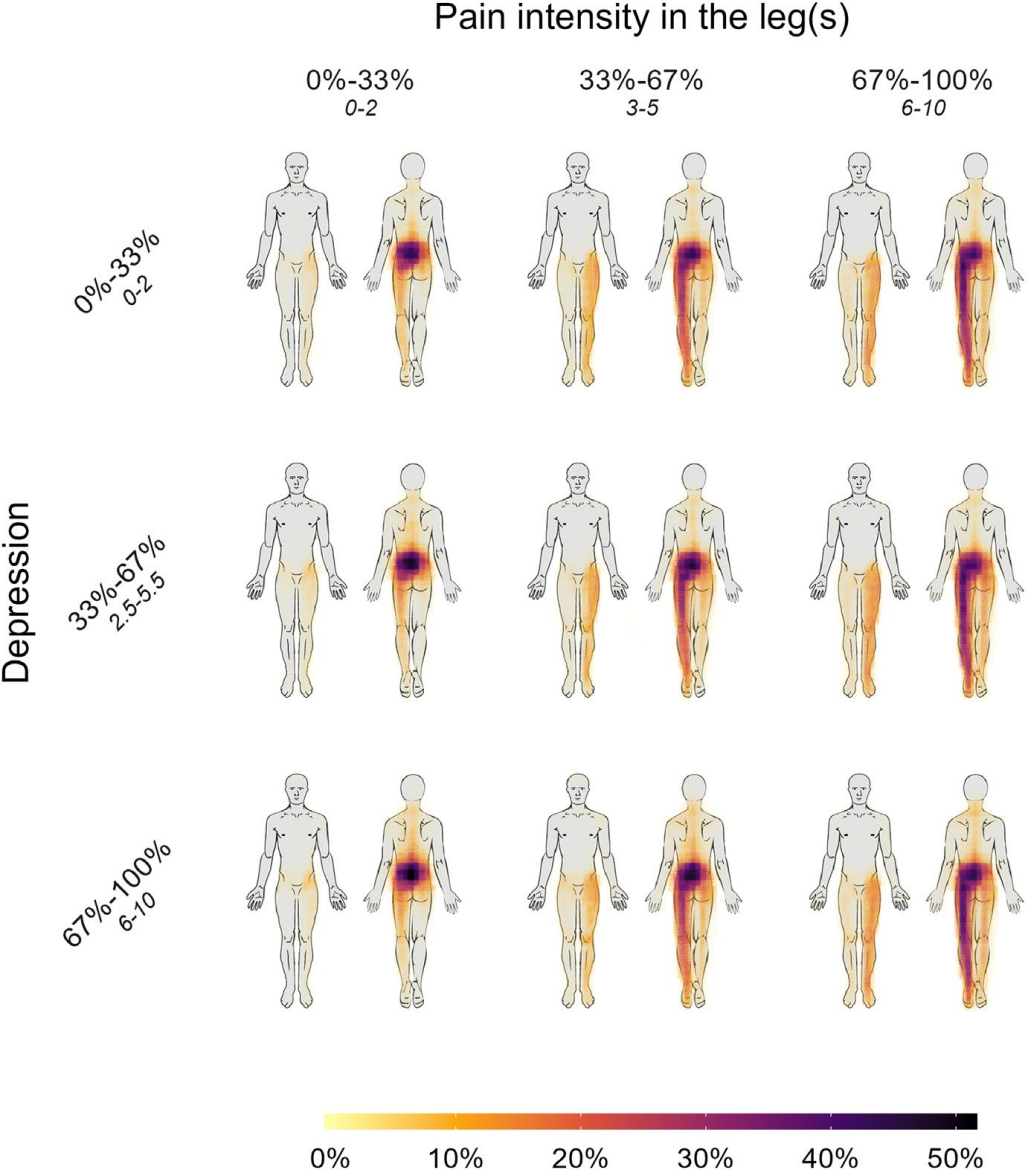
Aggregate pain drawings split by leg pain and depression. Each cell in the 3 × 3 grid represents a high transparency overlay of 1000 patient‐drawn pain drawings. Unilateral pain is always depicted on the left side of the body. The columns represent increasing leg pain scores from left to right, while the rows represent increasing depression scores from top to bottom. The range of the absolute scores is written below the percentage scores. The colours depict the percentage of patients within the cell who drew their pain in a given area. Comparing top to bottom within each of the columns (different grades of leg pain intensity), it can be seen that increasing depression scores only have a minor influence on the pain drawings. Contrary, comparing left to right within each of the rows (different grades of depression scores), it can be seen that increasing scores of pain intensity in the legs has a major influence on the pain drawings.

**FIGURE 3:**
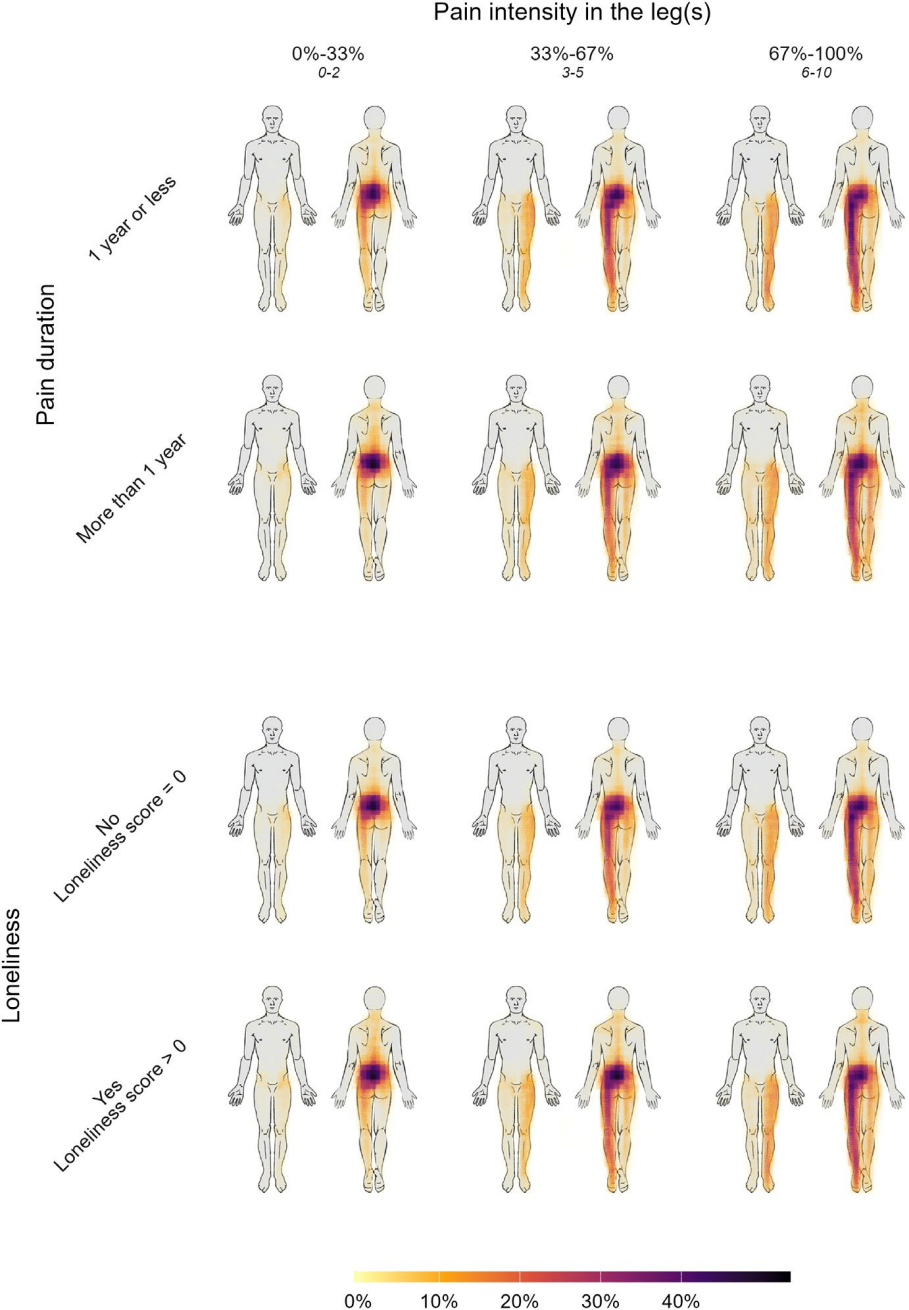
Uni‐ and Multivariable Regression Model Estimates. Each cell represents a high transparency overlay of 1000 patient‐drawn pain drawings. Unilateral pain is always depicted on the left side of the body. The columns represent increasing leg pain scores from left to right. The range of the absolute scores within each column is written below the percentage scores. The rows represent pain duration of more or less than a year and the absence or presence of loneliness. The colours depict the percentage of patients within each cell who drew their pain in a given area. Within each of the columns (different grades of leg pain intensity), it can be seen that longer pain duration and the presence of loneliness have some effect on the pain drawings. Specifically with pain in the upper body.

The aggregate pain drawings illustrate that the majority of the sample reported pain predominantly located in the lower back, frequently extending to involve varying degrees of unilateral or bilateral leg pain. The most substantial differences were observed with increasing scores of leg pain intensity, pain duration, pain‐related disability, and loneliness. In contrast, the visual differences in the pain drawings were relatively minor when patients with low and high levels of the other psychological measures (e.g. depression) were compared. Representative illustrations of these patterns are provided in Figure 1. These relationships were consistent across different scores of leg pain intensity, as illustrated in Figures 2 and 3 using depression, pain duration, and loneliness as examples (see supplementary material for the rest).

**FIGURE 4:**
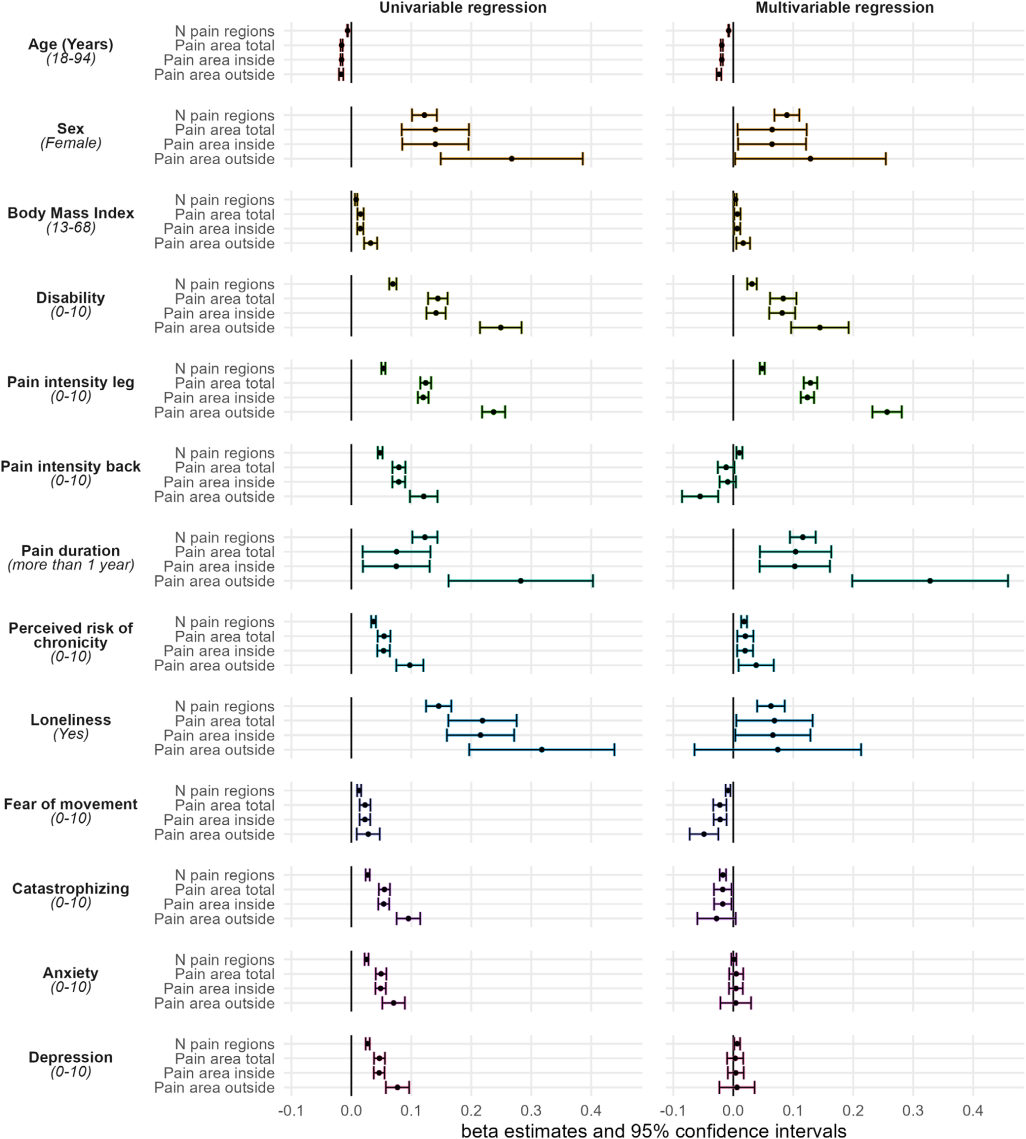
Aggregate pain drawings split by leg pain and pain duration. Each cell in the 3 × 2 grid represents a high transparency overlay of 1000 patient‐drawn pain drawings. Unilateral pain is always depicted on the left side of the body. The columns represent increasing leg pain scores from left to right, while the rows represent increasing pain duration from top to bottom. The range of the absolute leg pain scores is written below the percentage scores. The colours depict the percentage of patients within the cell who drew their pain in a given area.

We found statistically significant relationships between the independent variables and the four geometric pain area measures across all univariable regression models. Notably, these relationships were predominantly in a positive direction, except for age (Figure 4). Detailed results of these univariable analyses are provided in Table S1.

In the four multivariable regression models, which all included 13,576 complete cases, increasing pain intensity in the legs was generally the most influential factor, showing positive and statistically significant associations across all area measures, with beta estimates ranging from 0.12 to 0.25 (*p* < 0.001). Pain duration of 1 year or more exhibited positive associations with pain areas, with beta estimates ranging from 0.10 to 0.33 (*p* < 0.001). Pain‐related disability also showed positive and statistically significant associations (beta estimates ranging from 0.03 to 0.13, *p* < 0.001). In contrast, increasing age was consistently negatively and statistically significantly associated with all area measures (beta estimates ranging from −0.02 to −0.01, p < 0.001).

For the psychological distress measures, the relationships varied. Loneliness was positively and statistically significantly associated with ‘Pain Area Total,’ ‘Pain Area Inside,’ and ‘Number of Regions with Pain’ (beta estimates ranging from 0.06 to 0.07, *p* < 0.039), but did not have a statistically significant relationship with ‘Pain Area Outside’(*p* = 0.3). Fear of movement and catastrophizing were negatively and, in general, statistically significantly associated across all area measures (beta estimates ranging from −0.01 to −0.05). The relationships between anxiety and depression and the four pain area measures yielded some of the most minor beta estimates (ranging from −0.02 to 0.01) and were generally inconclusive, with *p*‐values predominantly above 0.4, except for a significant association between depression and the number of regions with pain (*p* = 0.009).

The direction and magnitude of the associations between the independent variables and the geometric area measures were consistent across all four multivariable models (See Table 2 and Figure 4 for further details). Despite similar beta estimates, pain intensity in the legs, measured on a 0–10 scale, showed a greater influence on pain area than the binary‐classified pain duration (Figure 3).

**TABLE 2:** Multivariable linear regression models showing the association between the four area measures and the independent variables.

	Pain area total (log changed)			Pain area inside (log changed)		
	Beta	95% CI	*p*	Beta	95% CI	*p*
Age (Years)	−0.02	−0.02, −0.02	<0.001	−0.02	−0.02, −0.02	<0.001
Sex						
Male	—	—		—	—	
Female	0.06	0.01, 0.12	0.027	0.06	0.01, 0.12	0.025
Body Mass Index	0.01	0.00, 0.01	0.010	0.01	0.00, 0.01	0.013
Disability	0.08	0.06, 0.11	<0.001	0.08	0.06, 0.10	<0.001
Pain intensity back	−0.01	−0.03, 0.00	0.089	−0.01	−0.02, 0.00	0.2
Pain intensity leg	0.13	0.12, 0.14	<0.001	0.12	0.11, 0.13	<0.001
Pain duration						
1 year or less	—	—		—	—	
More than 1 year	0.10	0.04, 0.16	<0.001	0.10	0.04, 0.16	<0.001
Perceived risk of chronicity	0.02	0.01, 0.03	0.003	0.02	0.01, 0.03	0.004
Loneliness						
No	—	—		—	—	
Yes	0.07	0.01, 0.13	0.034	0.07	0.00, 0.13	0.039
Fear of movement	−0.02	−0.03, −0.01	<0.001	−0.02	−0.03, −0.01	<0.001
Catastrophizing	−0.02	−0.03, 0.00	0.019	−0.02	−0.03, 0.00	0.017
Anxiety	0.00	−0.01, 0.02	0.4	0.00	−0.01, 0.02	0.5
Depression	0.00	−0.01, 0.02	0.6	0.00	−0.01, 0.02	0.5

	Pain area outside (log changed)			Nr. of regions with pain (log changed)		
	Beta	95% CI^a^	*p* ^b^	Beta	95% CI^a^	*p* ^b^
Age (Years)	−0.02	−0.03, −0.02	<0.001	−0.01	−0.01, −0.01	<0.001
Sex						
Male	—	—		—	—	
Female	0.13	0.00, 0.25	0.045	0.09	0.07, 0.11	<0.001
Body Mass Index	0.02	0.01, 0.03	0.005	0.00	0.00, 0.01	<0.001
Disability	0.14	0.10, 0.19	<0.001	0.03	0.02, 0.04	<0.001
Pain intensity back	−0.06	−0.09, −0.03	<0.001	0.01	0.01, 0.02	<0.001
Pain intensity leg	0.26	0.23, 0.28	<0.001	0.05	0.04, 0.05	<0.001
Pain duration						
1 year or less	—	—		—	—	
More than 1 year	0.33	0.20, 0.46	<0.001	0.12	0.09, 0.14	<0.001
Perceived risk of chronicity	0.04	0.01, 0.07	0.011	0.02	0.01, 0.02	<0.001
Loneliness						
No	—	—		—	—	
Yes	0.07	−0.06, 0.21	0.3	0.06	0.04, 0.09	<0.001
Fear of movement	−0.05	−0.07, −0.02	<0.001	−0.01	−0.01, 0.00	<0.001
Catastrophizing	−0.03	−0.06, 0.00	0.087	−0.02	−0.02, −0.01	<0.001
Anxiety	0.00	−0.02, 0.03	0.8	0.00	0.00, 0.01	0.7
Depression	0.01	−0.02, 0.04	0.7	0.01	0.00, 0.01	0.009

*Note*: This table presents beta coefficients from regression models with log‐transformed dependent variables. Exponentiating the beta estimates will therefore give the estimated percent change in pain area for a given predictor. For instance, transitioning from ‘No’ to ‘Yes’ for Loneliness is associated with an approximately 7.3% increase in total pain area (𝑒^0.07^ ≈1.0725). Similarly, a 3‐point increase in Disability is linked to a cumulative increase of about 27.1% in total pain area (𝑒^0.08*3^ ≈1.2712).

^a^ CI, confidence interval.

^b^ *p*, *p*‐value.

There was no multicollinearity in all multivariable models (Variance Inflation factor <5). Model residuals met the assumptions of normality and homoscedasticity for the ‘Area total,’Area inside,’ and ‘Nr. Of regions with pain’ models. However, the ‘Area outside’ model posed a challenge to the assumption of normality for the residuals, thus requiring further sensitivity analyses.

### 3.1 Sensitivity analysis

To model ‘Area outside,’ we employed alternative strategies to test the robustness of our findings. The alternative modelling strategies included zero‐inflated negative binomial regression, Poisson regression, and ordinal logistic regression—which yielded similar relationships in terms of direction and magnitude between the independent variables and the ‘Area outside’ measure as the log‐transformed multivariable linear regression model. However, assumptions for the alternative models were also not met (see supplementary material).

## 4 DISCUSSION

In this large‐scale observational study, we determined the relationships between various psychological and pain‐related variables and the geometric areas of PDPDs in a large cohort of patients with chronic LBP seen in a hospital setting. Our comprehensive examination of these relationships provides unique insights into how patients visually represent their pain experience in relation to psychological and pain constructs. High pain intensity, pain duration, and pain‐related disability were all strongly and consistently associated with larger geometric pain areas in PDPD, while the association between the psychological distress measures and the pain areas were generally weaker and less statistically significant.

Our univariable regression models initially corroborates the historical perspective, that larger areas or ‘expansion’ are linked to worse psychological functioning. Across all six psychological constructs: depression, anxiety, fear of movement, catastrophizing, loneliness, and perceived risk of chronicity we see positive and statistically significant associations with the four pain area measures. However, the narrative shifts in our multivariable regression analysis. In these models, the influence of psychological variables on pain area size becomes less pronounced. Loneliness and perceived risk of chronicity still show associations with larger pain areas, albeit to a lesser extent. The beta estimates for loneliness (0.06 to 0.07) appear similar to those for disability (0.03 to 0.14) and pain intensity in the legs (0.05 to 0.26) across the four multivariable models. However, it is important to note that loneliness is a dichotomous variable, while disability and pain intensity are measured on a continuous 0–10 scale, and their beta estimates, therefore, refer to one unit increases on these scales. Anxiety and depression were not associated with the area measures, while higher distress in ‘fear of movement’ and catastrophizing was associated with smaller pain areas.

Despite their initial positive associations in univariable analyses, ‘fear of movement’ and catastrophizing exhibit negative associations in the full multivariable models. This reversal suggests that the initial positive correlations may be driven by higher levels of pain intensity and disability, factors not controlled for in univariable regressions. In the comprehensive multivariable models, once these confounding factors are accounted for, the associations for ‘fear of movement’ and catastrophizing reverse. Although the beta estimates are small, these findings were unexpected and warrant further discussion. One plausible explanation is that these negative associations are artefacts of our analysis methods. Another speculative explanation is that patients with higher scores on ‘fear of movement’ and catastrophizing may adopt a more cautious and precise approach when depicting their pain in drawings. However, this interpretation is speculative and contrasts with the usual understanding of catastrophizing, which typically involves a magnification of pain. Given these uncertainties, future research should explore this relationship further. Additional studies should aim to replicate these findings using more comprehensive measures across different populations to determine the robustness of our findings and to understand the underlying mechanisms better.

Our study observed consistent directions and strengths in the associations of psychological and pain‐related variables across the four different geometric pain area measures. While the magnitude of the beta estimates varied slightly, no single area measure distinctly reflected worse psychological functioning in our population. Therefore, our data does not support the interpretation of drawing outside the body outline as a sign of intensified distress. Given that this metric is unlikely to provide reliable information on either physical or psychological health, we recommend that researchers and clinicians disregard this metric in future assessments.

Within a contemporary biopsychosocial framework, persistent pain is understood to be predisposed, precipitated, and perpetuated by salient psychosocial factors and neurobiological changes to the nervous system (Fillingim, 2017). Modern descriptors of such pain experiences include neuropathic, nociceptive, and nociplastic pain, with the latter describing an ill‐defined pain presentation in the absence of objective or consistent pathology (Fitzcharles et al., 2021). Historically, nociplastic pain presentations have been more strongly associated with maladaptive psychological functioning (Rief & Broadbent, 2007). In the present study, we hypothesized that excessive and expansive drawings both inside and outside the silhouette were associated with psychological distress, and that this methodology could serve as a proxy for subsequent psychological assessment. However, our findings did not support this hypothesis. The results indicate that PDPDs has limited utility in predicting psychological distress or potentially indicating a nociplastic pain presentation.

A recent study by Weßollek et al. (2021) concluded that although pain drawings exhibited correlations with anxiety and depression, they were not sufficiently reliable as standalone screening tools for these psychological outcomes (Weßollek et al., 2021). Their study, also employing multivariable linear regression, did identify a statistically significant relationship between pain drawing features and symptoms of anxiety and depression. However, while their multivariable models controlled for pain duration, they did not account for pain intensity or disability—two critical variables in our analysis. When these factors were included in our models, the associations between anxiety, depression, and pain area size had a small magnitude and were no longer statistically significant. This discrepancy highlights the importance of a more comprehensive model for analysing these relationships, as it suggests that observed relationships between psychological symptoms and pain drawing features may be confounded by physical symptoms such as pain intensity, pain duration, and disability.

Within LBP patients, the drawn pain areas appear to be significantly influenced by the presence and intensity of leg pain. This observation suggests that specific LBP diagnoses, particularly those associated with lower extremity pain, and the pathoanatomical basis of these diagnoses could be crucial factors in an individual's pain experience, representation, and communication of symptoms to caregivers, and further research should explore this.

The sample population in our study is a hospital‐based population of patients with LBP and likely represents a subset of patients with more severe LBP conditions. This severity could influence the extent and nature of pain depicted in their drawings as well as their levels of psychological distress. Additionally, some pathoanatomical conditions in the spine often result in leg pain and, therefore, increased pain areas within LBP patients. Such a pathoanatomical basis for increased pain areas might not be present in other non‐spine‐related pain conditions or populations. Thus, while our study offers valuable insights for LBP patient assessment in a hospital setting, extrapolating these results to other populations should be cautiously approached.

Thus, while PDPDs can be valuable in the clinical setting, they should be interpreted as part of a broader, multifaceted assessment, integrating other clinical and psychosocial information. When clinicians encounter a PDPD depicting a large area of pain, they should primarily consider this as an indicator of significant physical symptoms—pain intensity, duration, and disability. Although it may be tempting to link extensive pain depiction to worse psychological functioning, our cross‐sectional analysis does not support this approach. We recommend that clinicians focus more on the physical aspects indicated by the pain drawings and ensure a comprehensive evaluation by employing additional diagnostic tools aimed at assessing psychological health where necessary.

### 4.1 Study limitations

First, we used brief screening questionnaires with demonstrated concurrent validity comparable to full‐length questionnaires within a similar clinical population (Kent et al., 2014). While these instruments efficiently capture key psychological measures relevant to our study, we acknowledge that they may not encompass all psychological dimensions pertinent to the pain experience. The concise nature of these questionnaires, designed for broad clinical utility, might limit the detection of subtler aspects of psychological distress not covered by the major psychological constructs.

Second, was related to the scope of our examination. Our study focused solely on the size of the drawn areas, leaving out additional layers of information such as the specific pain locations, perceived sensations (e.g. numbness, burning, stabbing, pins & needles), and unique patient annotations (Ransford et al., 1976; Udén et al., 1988). Moreover, our sample's heterogeneity in LBP diagnoses may have further impacted the observed relationships. For instance, specific pain patterns may exist for conditions like lumbar spinal stenosis and lumbar disc herniation, which our study did not separately investigate. Therefore, while our findings contribute valuable insights into how the size of the drawn areas relates to psychological and clinical variables, they should be considered foundational.

Finally, our third limitation is related to the challenges we faced while modelling ‘Area Outside.’ Despite different statistical approaches, none could fully uphold the necessary assumptions for their respective model types. This adds a level of caution to the interpretation of the ‘Area Outside’ models. However, it is noteworthy that while the confidence intervals from this model were less precise, the direction and relative strengths of the independent variables were consistent with the findings in the three other models.

### 4.2 Future research and implications

A relevant extension of our work would be to investigate how specific clinical LBP diagnoses, such as lumbar disc herniation or lumbar spinal stenosis, might influence the characteristics of pain drawings. Studying the interplay between pain intensity and spatial distribution may yield valuable insights, especially in patients where these variables do not correlate. Longitudinal research focusing on the outcomes of patients with large pain areas could provide crucial information on the temporal aspects of pain management and the potential evolution of pain syndromes over time. Furthermore, repeating our analysis in different pain populations could validate and expand our findings, thereby increasing their applicability.

## 5 CONCLUSION

In this large sample of LBP patients from an outpatient public‐funded hospital unit specializing in adult spine pain syndromes, we found that increasing levels of leg pain intensity, pain duration, and pain‐related disability were consistently and significantly associated with larger geometric pain areas in PDPDs. Conversely, the associations between several psychological measures and the geometric pain areas exhibited varying directions and were notably weaker. Clinicians are encouraged to focus on the association of PDPDs with physical symptoms rather than psychological conditions during clinical assessments.

## AUTHOR CONTRIBUTIONS

**Steen Harsted**: Conceptualization, Methodology, Data Curation, Formal Analysis, Visualization, Writing —Original Draft Preparation. **Natalie H. S. Chang**: Software, Data Curation, Writing—Review & Editing. **Casper Nim**: Conceptualization, Methodology, Writing—Review & Editing. **James J. Young**: Conceptualization, Methodology, Writing—Review & Editing. **David T. McNaughton**: Conceptualization, Methodology, Writing—Review & Editing. **Søren O'Neill**: Software, Data Curation, Conceptualization, Methodology, Writing—Review & Editing.

## FUNDING INFORMATION

This research did not receive any specific grant from funding agencies in the public, commercial, or not‐for‐profit sectors.

## CONFLICT OF INTEREST STATEMENT

The authors declare that they have no conflicts of interest or financial disclosures to report that are relevant to the content of this manuscript.

## Supporting information


Appendix S1.

Data S1.

Data S2.

Table S1.
