# Supplementary material for: Exploring the association between patient‐drawn pain diagrams and psychological and physical health variables: A large‐scale study of patients with low back pain
Source: Eur J Pain. 2024 Aug 7;29(2):e4711. doi: 10.1002/ejp.4711 (PMC11671319; doi:10.1002/ejp.4711)
Supplement: Supplementary file 1 — Appendix S1. [file EJP-29-0-s001.pdf]

Items	answers / Labels	question type	Variable name
Patient ID		placeholder.question	PatientID
Patient sex	(0) Female<br>(1) Male	placeholder.question	patientSex
Patient age on the date of consultation		placeholder.question	age_at_visit
Patient BMI calculated from weight and height self-reported		calculated integer question	BMI
Paindrawing - free hand - SVG format		hidden.text.question	paindrawing
Pain duration	Approximately, when was the onset of your current back or leg pain? (date)<br><br>Choose a date in the calendar or write the date in the space in the format year-month-date (yyyy-mm-dd).	date	pain_duration
Current backpain	No pain to worst pain - VAS skala 0-10 point	0-10 Radio Buttons	painnow
Typical backpain the last 14 days	No pain to worst pain - VAS skala 0-10 point	0-10 Radio Buttons	typicalpain
Worst backpain the last 14 days	No pain to worst pain - VAS skala 0-10 point	0-10 Radio Buttons	worstpain
Current legpain	No pain to worst pain - VAS skala 0-10 point	0-10 Radio Buttons	legpainnow
Typical legpain (iskias) the last 14 days	No pain to worst pain - VAS skala 0-10 point	0-10 Radio Buttons	typical_legpain
Worst legpain (iskias) the last 14 days	No pain to worst pain - VAS skala 0-10 point	0-10 Radio Buttons	worst_legpain
Do you feel anxious?	Not at all - to a large extent 0-10 point skala	1-10 Radio buttons w/Legend	anxiety
Do you feel lonely?	Not at all - to a large extent 0-10 point skala	1-10 Radio buttons w/Legend	socialisolation
When I feel pain, they are horrible and I feel like it will never get better.	Not at all - to a large extent 0-10 point skala	1-10 Radio buttons w/Legend	catastrophisation1
When I feel pain, I feel like I can not handle it anymore.	Not at all - to a large extent 0-10 point skala	1-10 Radio buttons w/Legend	catastrophisation2
catastrophisation score (mean of item nr 18 and 19)		calculated score	catastro_mean
"Physical activity can damage my back."	Not at all - to a large extent 0-10 point skala	1-10 Radio buttons w/Legend	fearaktiv1
"I should refrain from physical activity that (possibly) can aggravate my pain."	Not at all - to a large extent 0-10 point skala	1-10 Radio buttons w/Legend	fearaktiv2
fear of activity score (mean of item nr 21 and 22)		calculated score	fearaktiv_mean
How big do you think the risk is that your current pain will be long lasting?	Not at all - to a large extent 0-10 point skala	1-10 Radio buttons w/Legend	riskpersist
Over the past month, have you often felt sad, depressed, or had a sense of hopelessness?	Not at all - to a large extent 0-10 point skala	1-10 Radio buttons w/Legend	depression1
During the past month, have you felt bothered by having decreased interest or joy in doing something?	Not at all - to a large extent 0-10 point skala	1-10 Radio buttons w/Legend	depression2
depression score (mean of item nr 25 and 26)		calculated score	depression_mean
ODI /NDI (10 items) functionscore (disability)	Sum of scores / (max score - missing items * max item score) * 100	calculated score	functionscore
Patient type/ primary area of pain	LBP - lumbal MBPNP - cervical and thoracal	placeholder.question	patienttype
Calculated paindrawing area - total area (inside, outside and overlaps)	polygons generated with Søren's method - simple polygons/convexhull	calculated sum	Area_poly
Calculated paindrawing area - inside paindrawing with overlaps	polygons generated with Søren's method - simple polygons/convexhull	calculated sum	Area_intersect
Calculated paindrawing area - outside paindrawing	polygons generated with Søren's method - simple polygons/convexhull	calculated sum	Area_ouside

Total area (item 36) minus overlap	polygons generated with Søren's method - simple polygons/convexhull	calculated	Area_without_overlap_fullPoly
Calculated paindrawing area - inside paindrawing without overlaps	polygons generated with Søren's method - simple polygons/convexhull	calculated	Area_without_overlap_onlyindrawing
Number of anatomical regions marked (total 46)		sum of anatomical regions	N_Regions
Number of polygons generated	polygons generated with Søren's method - simple polygons/convexhull	calculated	N_poly
Number of singlepoints in paindrawing (stroke of only one coordinate pair)		calculated	N_singlepoints
Anatomical region marked	(1) No<br>(2) Yes	binary	R1 - R46
