# Supplementary material for: Exploring the association between patient‐drawn pain diagrams and psychological and physical health variables: A large‐scale study of patients with low back pain
Source: Eur J Pain. 2024 Aug 7;29(2):e4711. doi: 10.1002/ejp.4711 (PMC11671319; doi:10.1002/ejp.4711)
Supplement: Supplementary file 2 — Data S1. [file EJP-29-0-s003.pdf]

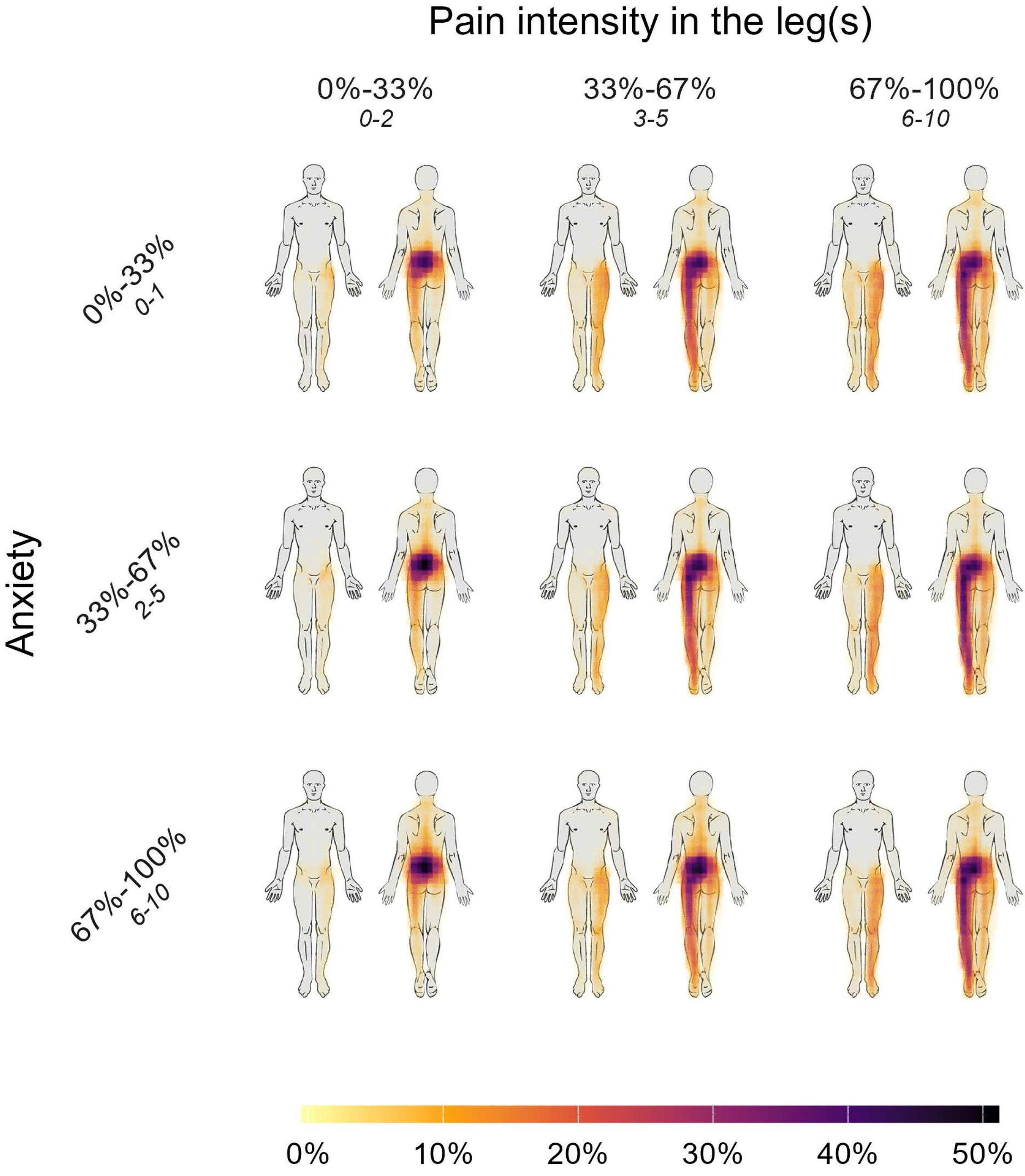
Pain intensity in the leg(s)
0%-33%
0-2
33%-67%
3-5
67%-100%
6-10
Anxiety
0%-33%
0-1
33%-67%
2-5
67%-100%
6-10
0%
10%
20%
30%
40%
50%

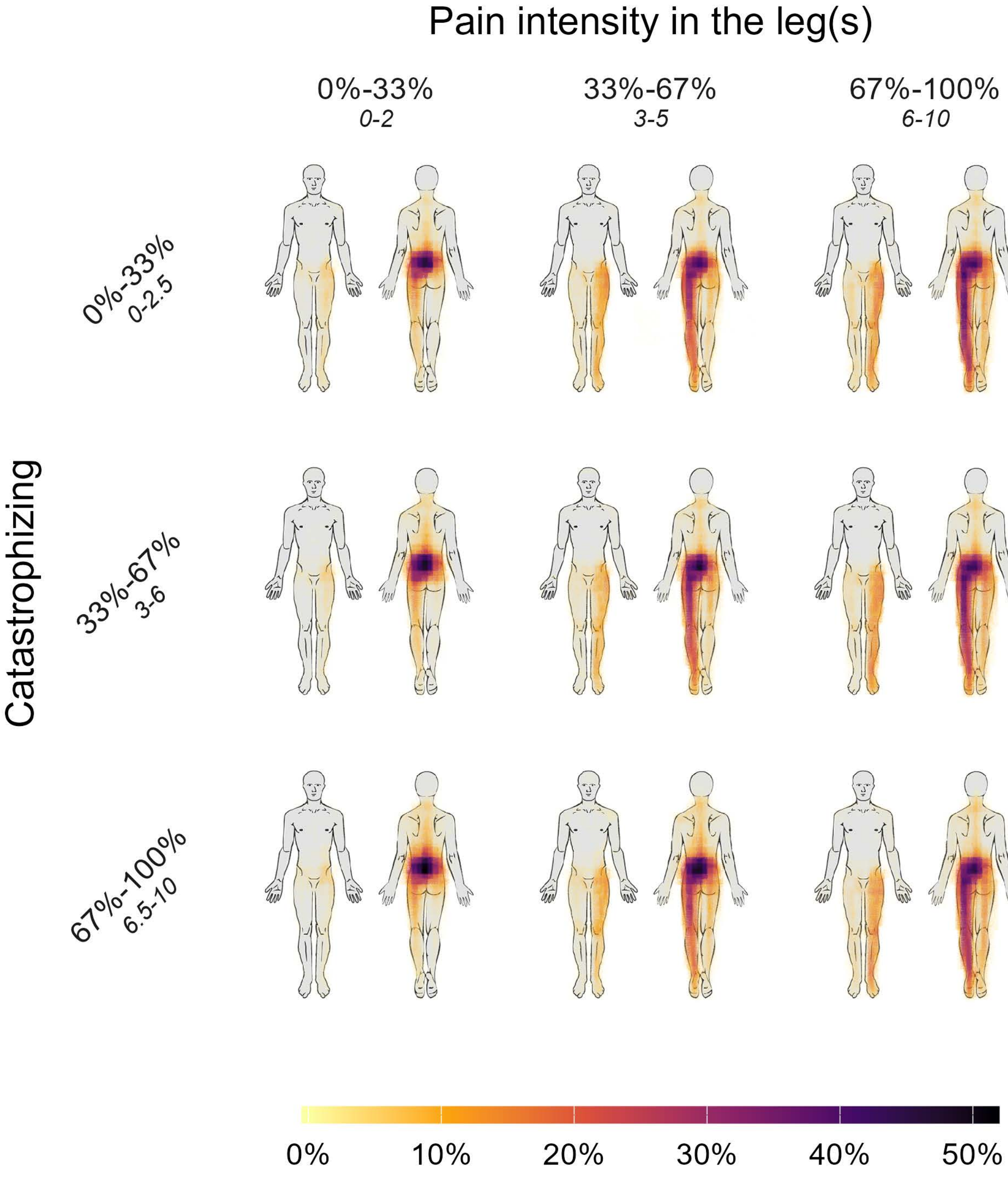

Pain intensity in the leg(s)
0%-33%
0-2
33%-67%
3-5
67%-100%
6-10
Catastrophizing
0%-33%
0-2.5
33%-67%
3-6
67%-100%
6.5-10
0%
10%
20%
30%
40%
50%

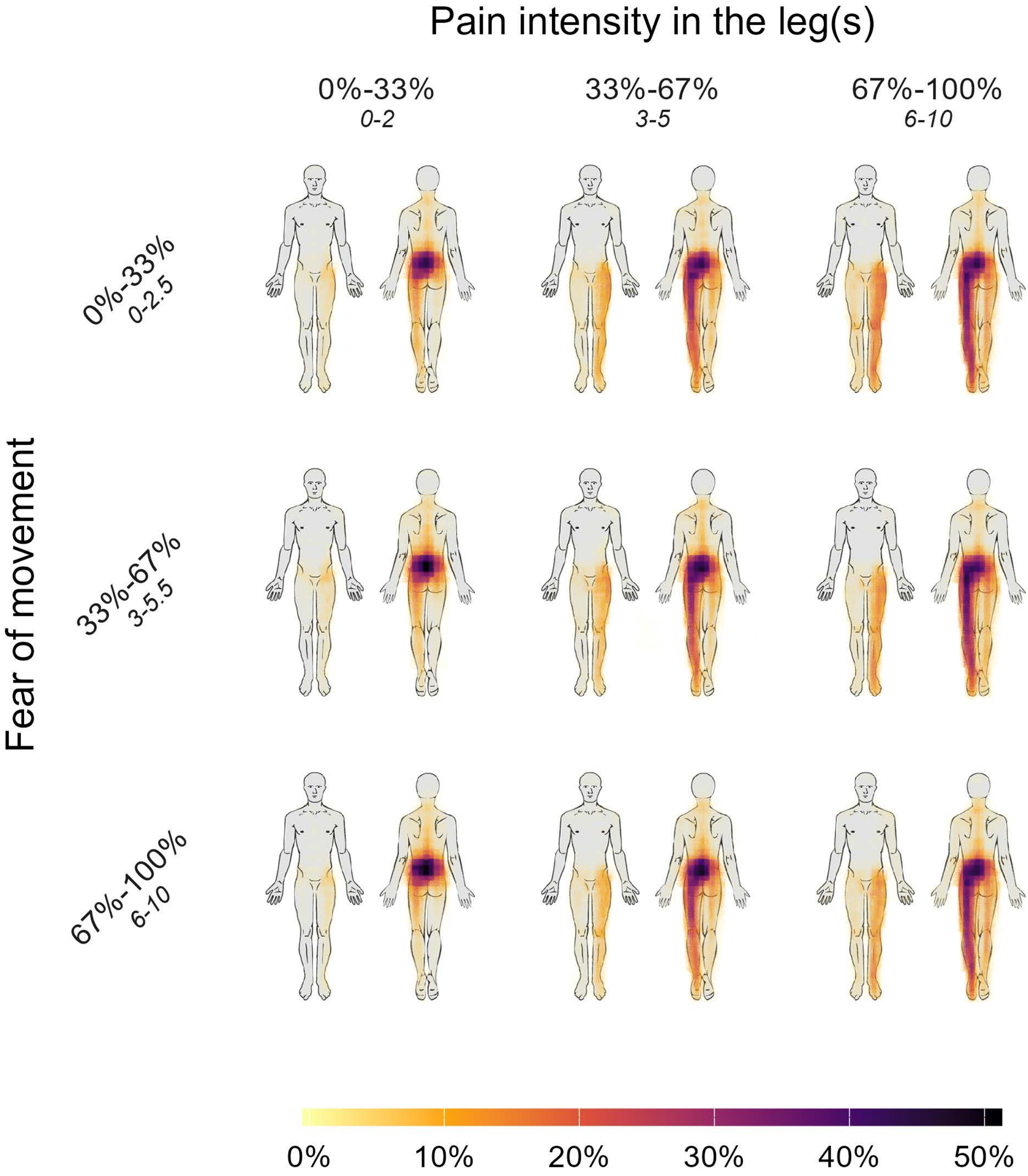

Pain intensity in the leg(s)
0%-33%
0-2
33%-67%
3-5
67%-100%
6-10
Fear of movement
0%-33%
0-2.5
33%-67%
3-5.5
67%-100%
6-10
0%
10%
20%
30%
40%
50%

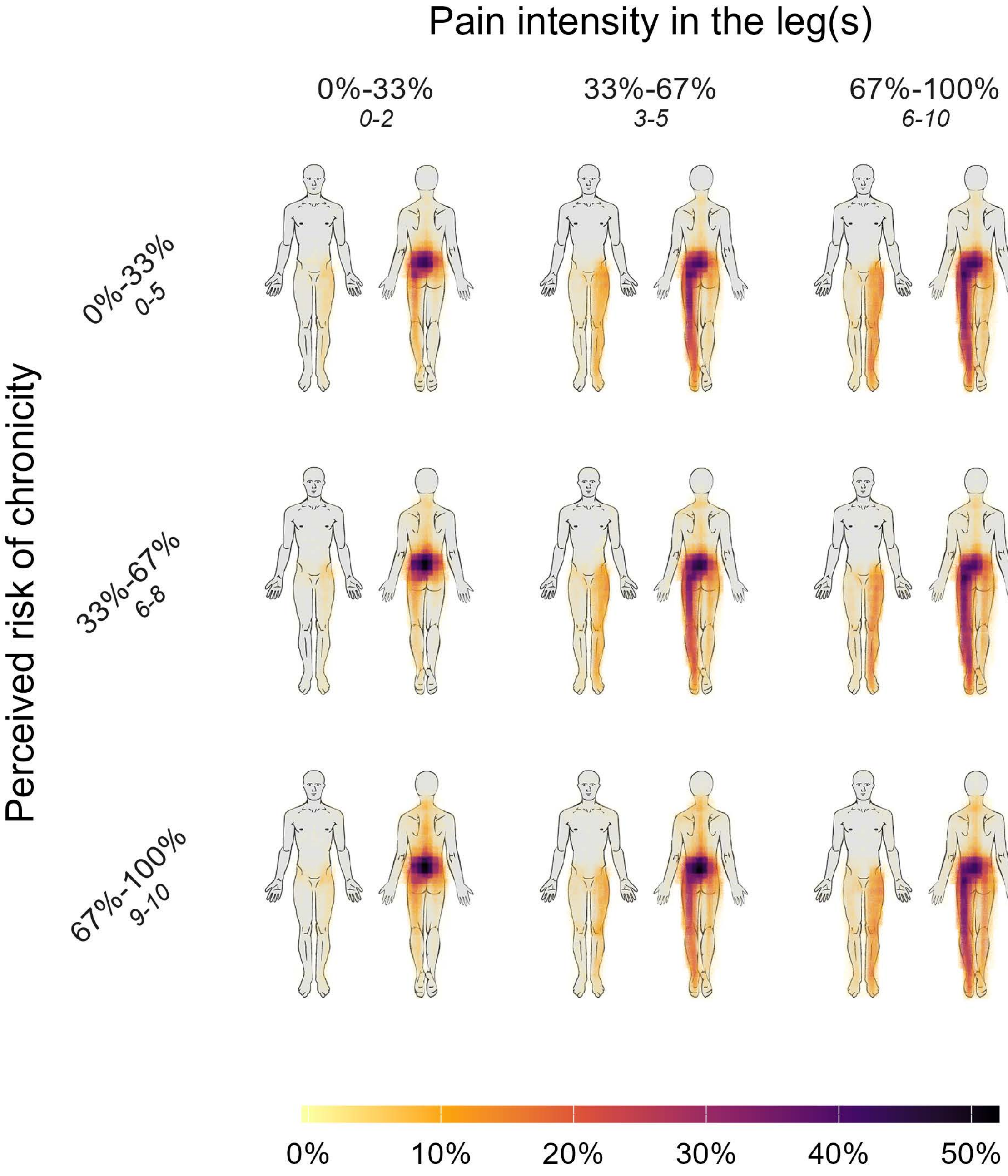
Pain intensity in the leg(s)
0%-33%
0-2
33%-67%
3-5
67%-100%
6-10
Perceived risk of chronicity
0%-33%
0-5
33%-67%
6-8
67%-100%
9-10
0%
10%
20%
30%
40%
50%
