# Supplementary material for: Exploring the association between patient‐drawn pain diagrams and psychological and physical health variables: A large‐scale study of patients with low back pain
Source: Eur J Pain. 2024 Aug 7;29(2):e4711. doi: 10.1002/ejp.4711 (PMC11671319; doi:10.1002/ejp.4711)

Log transformations of the four area measures

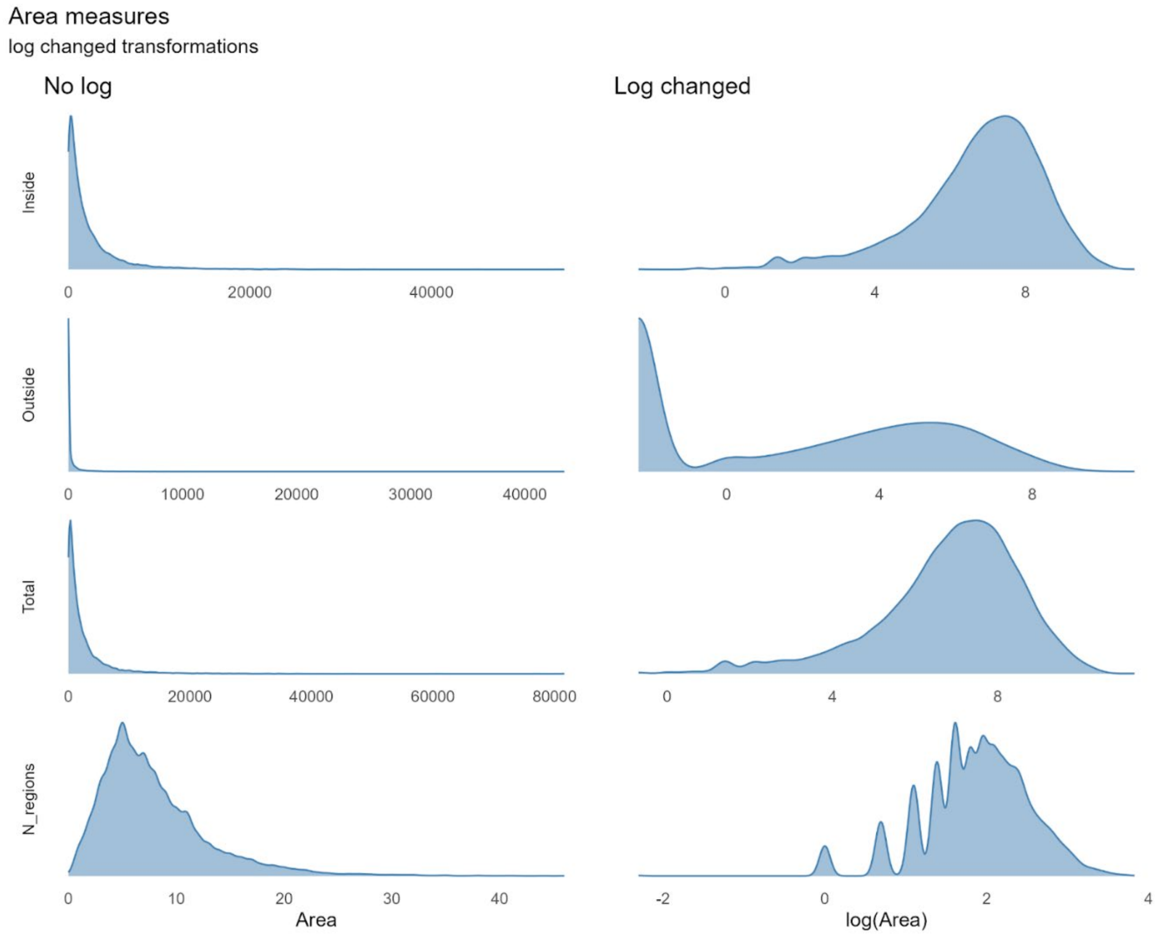

Note: To avoid the introduction of NA values, 0 values were changed to 0.1 before log-transformation.

Transformation of the loneliness variable from continuous to categorical.

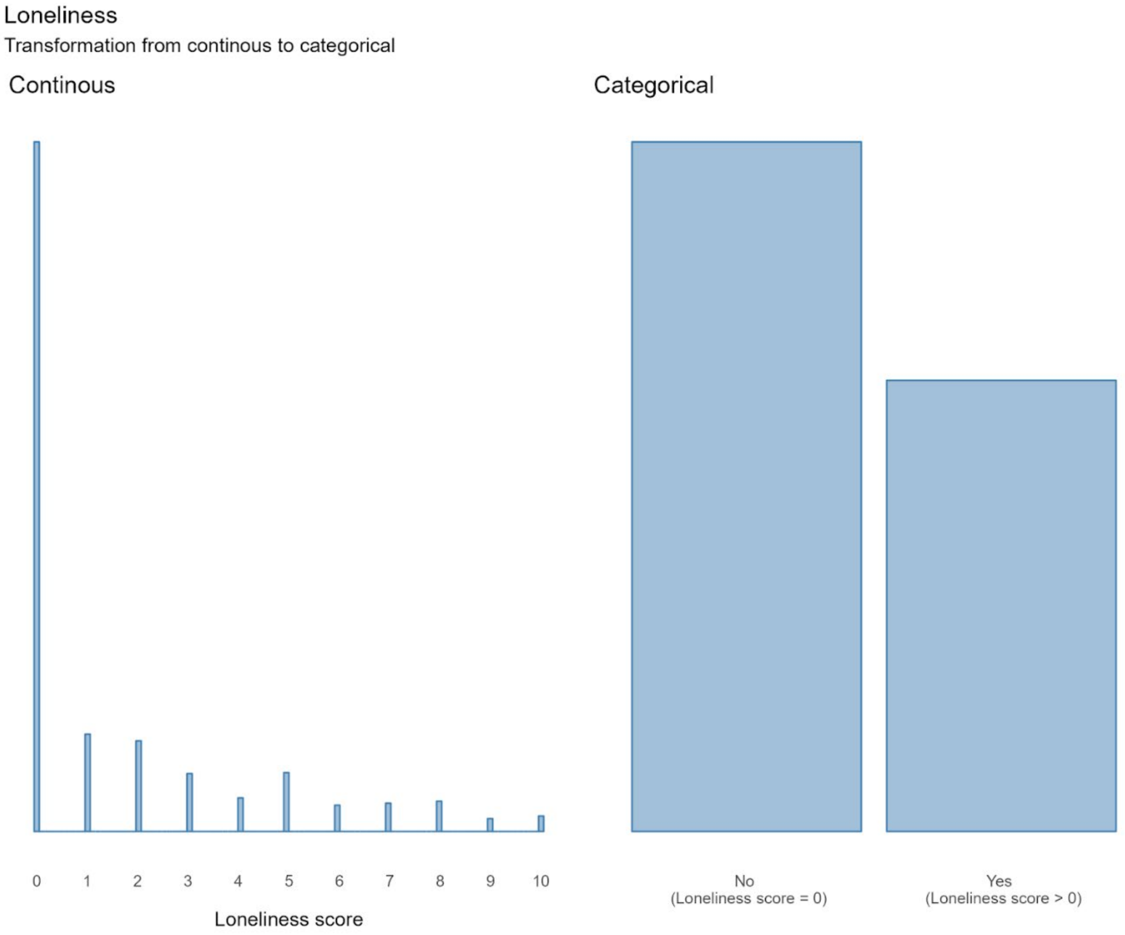

Transformation of the pain duration variable from continuous to categorical.

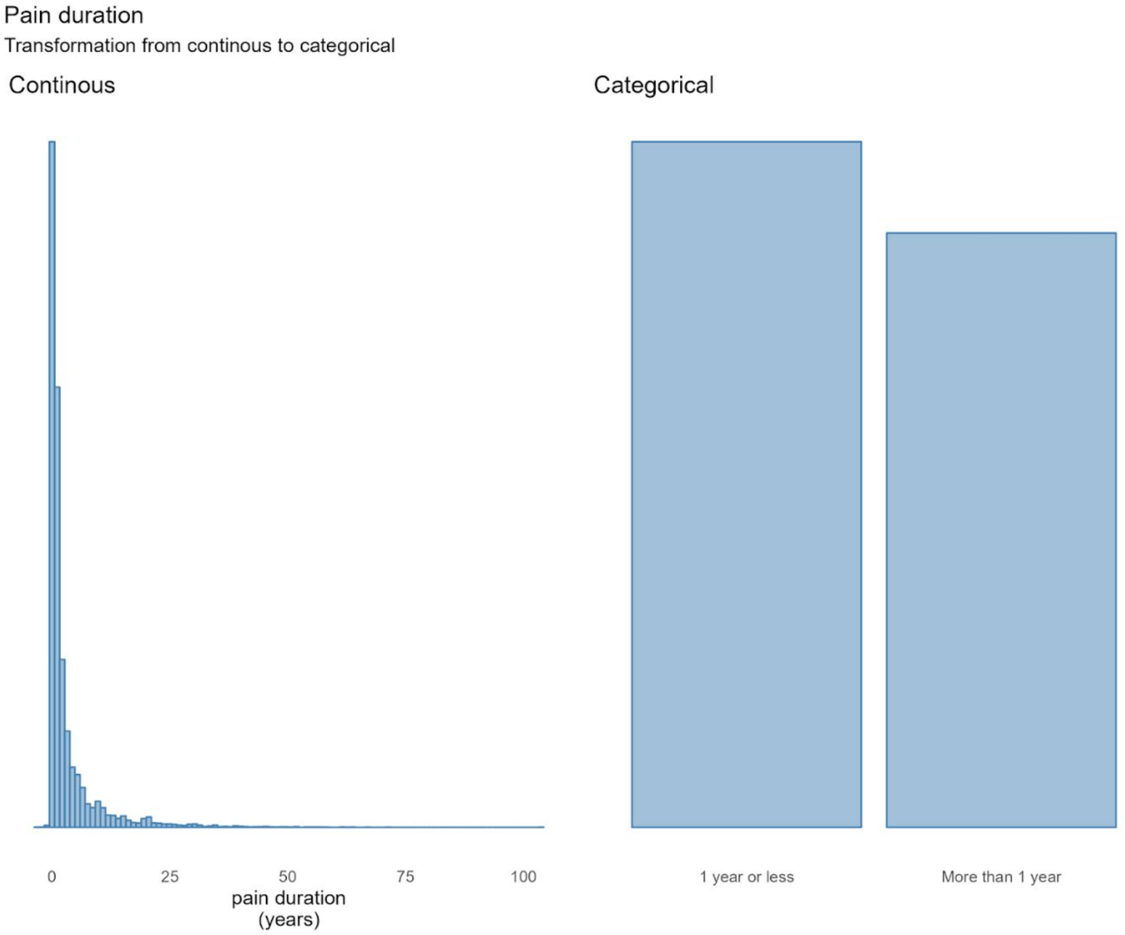

Supplement: Supplementary file 3 — Data S2. [file EJP-29-0-s004.pdf]
