# Supplementary material for: Exploring the association between patient‐drawn pain diagrams and psychological and physical health variables: A large‐scale study of patients with low back pain
Source: Eur J Pain. 2024 Aug 7;29(2):e4711. doi: 10.1002/ejp.4711 (PMC11671319; doi:10.1002/ejp.4711)
Supplement: Supplementary file 4 — Table S1. [file EJP-29-0-s002.docx]

| Supplementary table 1.  Linear univariable regression models modeling the association between the four area measures and the included independent variables. | | | | | | |
| --- | --- | --- | --- | --- | --- | --- |
|  | **Total** | | | **Inside** | | |
|  | **Beta** | **95% CI***^1^* | **p-value** | **Beta** | **95% CI***^1^* | **p-value** |
| Age (Years) | -0.02 | -0.02, -0.01 | <0.001 | -0.02 | -0.02, -0.01 | <0.001 |
| Sex |  |  |  |  |  |  |
| *Male* | — | — |  | — | — |  |
| *Female* | 0.14 | 0.08, 0.20 | <0.001 | 0.14 | 0.08, 0.20 | <0.001 |
| Body Mass Index | 0.02 | 0.01, 0.02 | <0.001 | 0.01 | 0.01, 0.02 | <0.001 |
| Disability | 0.14 | 0.13, 0.16 | <0.001 | 0.14 | 0.13, 0.16 | <0.001 |
| Pain intensity back | 0.08 | 0.07, 0.09 | <0.001 | 0.08 | 0.07, 0.09 | <0.001 |
| Pain intensity leg | 0.12 | 0.12, 0.13 | <0.001 | 0.12 | 0.11, 0.13 | <0.001 |
| Pain duratoin |  |  |  |  |  |  |
| *1 year or less* | — | — |  | — | — |  |
| *More than 1 year* | 0.08 | 0.02, 0.13 | 0.009 | 0.07 | 0.02, 0.13 | 0.008 |
| Perceived risk of chronicity | 0.05 | 0.04, 0.07 | <0.001 | 0.05 | 0.04, 0.06 | <0.001 |
| Loneliness |  |  |  |  |  |  |
| *No* | — | — |  | — | — |  |
| *Yes* | 0.22 | 0.16, 0.28 | <0.001 | 0.22 | 0.16, 0.27 | <0.001 |
| Fear of movement | 0.02 | 0.01, 0.03 | <0.001 | 0.02 | 0.01, 0.03 | <0.001 |
| Catastrophizing | 0.06 | 0.05, 0.06 | <0.001 | 0.05 | 0.04, 0.06 | <0.001 |
| Anxiety | 0.05 | 0.04, 0.06 | <0.001 | 0.05 | 0.04, 0.06 | <0.001 |
| Depression | 0.05 | 0.04, 0.06 | <0.001 | 0.05 | 0.04, 0.06 | <0.001 |
|  | **Outside** | | | **N regions with pain** | | |
|  | **Beta** | **95% CI***^1^* | **p-value** | **Beta** | **95% CI***^1^* | **p-value** |
| Age (Years) | -0.02 | -0.02, -0.01 | <0.001 | -0.01 | -0.01, -0.01 | <0.001 |
| Sex |  |  |  |  |  |  |
| *Male* | — | — |  | — | — |  |
| *Female* | 0.27 | 0.15, 0.39 | <0.001 | 0.12 | 0.10, 0.14 | <0.001 |
| Body Mass Index | 0.03 | 0.02, 0.04 | <0.001 | 0.01 | 0.01, 0.01 | <0.001 |
| Disability | 0.25 | 0.21, 0.28 | <0.001 | 0.07 | 0.06, 0.08 | <0.001 |
| Pain intensity back | 0.12 | 0.10, 0.14 | <0.001 | 0.05 | 0.04, 0.05 | <0.001 |
| Pain intensity leg | 0.24 | 0.22, 0.26 | <0.001 | 0.05 | 0.05, 0.06 | <0.001 |
| Pain duratoin |  |  |  |  |  |  |
| *1 year or less* | — | — |  | — | — |  |
| *More than 1 year* | 0.28 | 0.16, 0.40 | <0.001 | 0.12 | 0.10, 0.14 | <0.001 |
| Perceived risk of chronicity | 0.10 | 0.07, 0.12 | <0.001 | 0.04 | 0.03, 0.04 | <0.001 |
| Loneliness |  |  |  |  |  |  |
| *No* | — | — |  | — | — |  |
| *Yes* | 0.32 | 0.20, 0.44 | <0.001 | 0.15 | 0.12, 0.17 | <0.001 |
| Fear of movement | 0.03 | 0.01, 0.05 | 0.004 | 0.01 | 0.01, 0.02 | <0.001 |
| Catastrophizing | 0.10 | 0.08, 0.11 | <0.001 | 0.03 | 0.02, 0.03 | <0.001 |
| Anxiety | 0.07 | 0.05, 0.09 | <0.001 | 0.03 | 0.02, 0.03 | <0.001 |
| Depression | 0.08 | 0.06, 0.10 | <0.001 | 0.03 | 0.02, 0.03 | <0.001 |
| *^1^* CI = Confidence Interval | | | | | | |
